# Clinical Characteristics and Prevalence of Celiac Disease in a Large Cohort of Type 1 Diabetes from Saudi Arabia

**DOI:** 10.3390/medicina60121940

**Published:** 2024-11-25

**Authors:** Mohammed Hakami, Saeed Yafei, Abdulrahman Hummadi, Raed Abutaleb, Abdullah Khawaji, Yahia Solan, Turki Aljohani, Ali Jaber Alhagawy, Amer Al Ali, Shakir Bakkari, Morghma Adawi, Maram Saleh, Sayidah Zaylaee, Rashad Aref, Khaled Tahash, Ebrahim Haddad, Amnah Hakami, Mohammed Hobani, Ibrahem Abutaleb

**Affiliations:** 1 Adult Endocrinology and Diabetes Department, Jazan Endocrinology & Diabetes Center, Ministry of Health, Jazan 82723, Saudi Arabia; 2 Endocrinology Department, Faculty of Medicine and Health Sciences, Taiz University, Taiz 6803, Yemen; 3 Pediatric Endocrinology, Jazan Endocrinology & Diabetes Center, Ministry of Health, Jazan 82723, Saudi Arabia; 4 Gastroenterology Department, King Saud Medical City, Riyadh 11421, Saudi Arabia; 5 Nursing Department, Ministry of Health, Jazan 45142, Saudi Arabia

**Keywords:** celiac disease, gluten enteropathy, type 1 diabetes, Saudi Arabia

## Abstract

*Background and Objectives*: The link between celiac disease (CD) and type 1 diabetes (T1D) has been well-documented in the medical literature and is thought to be due to a shared genetic predisposition in addition to environmental triggers. This study aimed to determine the seroprevalence and biopsy-proven CD (PBCD) prevalence in individuals with T1D from Saudi Arabia and identify their clinical characteristics and the impact on glycemic control. *Materials and Methods*: A total of 969 children and adolescents with confirmed T1D were investigated. Prospective and retrospective data were collected to include clinical, anthropometric, and biochemical data. Total IgA and anti-TTG-IgA antibodies were screened to detect seropositive cases. Upper intestinal endoscopy and biopsy were performed to find BPCD. *Results*: The seroprevalence of CD was 14.6% (141/969), while BPCD prevalence was 7.5%. Females had a higher prevalence than males: 17.8% vs. 9.8%, *p* < 0.001. The CD group had lower HbA1c and more frequent hypoglycemia than the seronegative group. *Conclusions*: This study highlighted the high prevalence of CD in T1D Saudi patients. CD has multiple effects on glycemic control, growth, and puberty in children and adolescents with T1D. We emphasize the importance of early screening for CD at the time of diabetes diagnosis and periodically after that or if any atypical features present, especially anemia, growth delay, underweight, or frequent hypoglycemia.

## 1. Introduction

Celiac disease (CD) is a common autoimmune enteropathy that is characterized by a specific serological and histological profile triggered by gluten ingestion in a genetically predisposed individual [1]. Clinical presentation of CD is classified into classic, non-classic, subclinical, potential, and refractory types [2]. Classic CD is more common in children and mainly presents with intestinal manifestations like diarrhea, anorexia, abdominal distention, vitamin deficiencies, iron deficiency anemia, and failure to thrive. The non-classical forms are also common in children and mostly present with symptoms of abdominal pain, anemia, and short stature, while adults are mostly asymptomatic [2]. Patients with untreated CD may be presented with complications like osteoporosis, neurologic disorders, as well as enteropathy-associated T-cell lymphoma and adenocarcinoma of the jejunum [3].

Type 1 diabetes mellitus (T1DM) is an autoimmune disease characterized by an absolute deficiency of insulin secretion. The link between CD and type 1 diabetes has been well documented in the medical literature and is thought to be due to a shared genetic predisposition between the two conditions in addition to environmental triggers [3]. The incidence and prevalence of CD and T1D are rapidly increasing in children and adolescents. Recent estimates indicated that there are about 500,000 new cases of T1D discovered annually over the world; thus, the prevalence of T1D is increasing by 0.34% annually [4]. Similarly, the incidence of CD has been significantly raised in the last 30 years, most probably due to changes in environmental and dietary factors [5]. From epidemiological views, cross-sectional and longitudinal studies in T1D children and adolescents estimated that CD prevalence ranges from 1.6% to 16.4% worldwide [6]. In the general population, the seroprevalence of CD was 1.1% to 1.6% of all ages compared with 0.5–0.9% of biopsy-confirmed CD [7].

While CD and T1D have different clinical manifestations, they share similar genetic backgrounds and autoimmune mechanisms, highlighting the complex relationship between genetics and the environment in developing both conditions [8]. The enteropathy associated with celiac disease results from the presentation of gliadin peptide fragments by antigen-presenting cells via human leukocyte antigen (HLA) proteins. About 95% of individuals with CD carry specific variants of the human leukocyte antigen (HLA), particularly HLA-DR3/DQ2 and HLA-DQ8, to a lesser extent. Similarly, about 30–50% of patients with TID are DR3/DR4 heterozygotes, which confers the highest diabetes risk [8]. Additionally, 90% of individuals with T1D have either DQ2 or DQ8 compared with 40% of the general population [9]. HLA-DQ2 and DQ8 loci are the most important determinants of T1D susceptibility. T1D individuals who are homozygote for HLA-DR3/DQ2 carry a 33% risk for the presence of transglutaminase autoantibodies [10]. Despite this genetic background, T1D and CD have environmental and dietary triggers initiating autoimmune pathogenicity.

The coexistence of CD and T1D presents unique challenges for individuals with both conditions. Management of T1D in people with CD can be challenging. Individuals with both conditions must adhere to a strict gluten-free diet to manage celiac disease while also monitoring their blood sugar levels and administering insulin for T1D. Likewise, the dietary restrictions for celiac disease may impact blood sugar control in individuals with type 1 diabetes. Furthermore, in patients with T1D, the coexistence of CD not only affects intestinal absorption of nutrients, calcium, and skeletal metabolism but also may contribute to the higher risk of cardiovascular incidents and exaggerated complications, such as nephropathy, retinopathy, and neuropathy [11]. Either symptomatic or not, CD diagnosis can be screened by serologic investigations for serum IgA levels and specific autoantibodies, including anti-endomysium (EMA), anti-tissue transglutaminase (tTG), and anti-deamidated peptides of gliadin (DGP). CD confirmation small intestine endoscopy and biopsy to detect pathological enteropathy.

Saudi Arabia is one of the top 10 countries with the highest prevalence and incidence of type 1 diabetes in children and adolescents below the age of 20 [12]. Worldwide, and in Saudi Arabia, there is a growing concern about the prevalence of celiac disease in individuals with type 1 diabetes. The national prevalence of CD in the general population of Saudi Arabia is unknown, but epidemiological studies estimated CD prevalence between 1.5% and 2.2%, making it one of the highest seroprevalence rates in the world [13,14]. The high prevalence of T1D and CD in Saudi Arabia is not fully understood and cannot only be related to genetic factors; environmental and dietary factors might also be involved. Regarding the prevalence of CD in T1D, studies on children and adolescents reported diverse results, where the rates ranged from 7.1 to 24.5% in different regions of Saudi Arabia, which is also higher than the prevalence in Western countries [15,16,17]. The Saudi guidelines for the management of T1D in children recommend tests for celiac disease within one year of T1D diabetes, even in the absence of classic symptoms, and the test should be repeated within 4–5 years or at any time with clinical reasoning [18].

The objectives of this study were to estimate the seroprevalence and biopsy-proven prevalence (PBCD) in individuals with T1D, identify their clinical presentation, and assess the impact on glycemic control and implications for clinical practice.

## 2. Materials and Methods

### 2.1. Study Design and Patients

Jazan Endocrinology and Diabetes Center (JEDC) is a high-volume multidisciplinary care center in southwestern Saudi Arabia. This referral center provides care to more than 13,400 diabetic patients, about 16.8% of whom have T1D. This study is a mixed retrospective and prospective study that included 969 children and adolescents with confirmed T1D who attended the pediatric and adolescent endocrinology clinics between March 2020 and March 2023. Prospective data for this study were collected during the patient’s clinical visits, while retrospective data were collected from electronic medical records.

Inclusion criteria included all T1D patients between the ages of 2 and 18 years. T1D was confirmed by clinical profiling, C-peptide, and T1D autoantibodies. Patients with infrequent visits or deficiency of medical records were managed as new cases to complete their medical profile before enrollment in the study. Patients with type 2 diabetes, uncategorized diabetes, diabetes other than T1D, and those who refused to participate in the study were excluded.

Sociodemographic and T1D-related information included age, sex, weight, height, age of diagnosis, duration of diabetes, and age of CD diagnosis. Clinical, anthropometric, and biochemical data were recorded. Body mass index (BMI) (kg/m^2^) was calculated from weight (kg) and height (m) and adjusted for age and sex. Those with a standing height below 2.5 SD or the 5th percentile for chronological age were considered short. Males above 14 years or females above 13 years without the appearance of signs of puberty are considered delayed puberty.

Glycemic control was assessed by HbA1c and ambulatory blood glucose profile (AGP). Total insulin dose (unit/kg/day), frequency, and severity of hypoglycemia from self-monitoring records and AGP were recorded. Other biochemical data included hemoglobin level, liver function test, serum iron, ferritin, vitamin D, and calcium level. In this study, if CD was already diagnosed before the beginning of the study, their medical records were retrospectively investigated for clinical, serological, and pathological data to be included in the prevalence rates.

### 2.2. Screening for CD

Intestinal and extraintestinal signs and symptoms related to CD were screened. Intestinal symptoms included chronic abdominal pain, diarrhea, constipation, abdominal distension, weight loss, vomiting, and anorexia. Extraintestinal symptoms included delayed puberty, stunted growth, iron-deficiency anemia, vitamin D levels, and liver transaminases.

According to ESPGHAN criteria for diagnosis of CD [19], serologic screening for CD was performed with measurement of total IgA and anti-tissue transglutaminase (anti-tTG-IgA). Anti-tTG IgA has high sensitivity, specificity, and positive predictive value and is the most reliable test for CD screening in people with normal serum IgA levels [20]. In this study, anti-EMA-IgA was requested for patients with low positive Anti-tTG IgA.

The cut-off value of anti-TTG-IgA ≥ 20 IU/mL is considered positive, as per the lab reference range. The seropositive group included any patient with positive anti-tTG-IgA. So, we divided the seropositive group into two subgroups.

Patients with anti-tTG Ab above 10× ULN.

Patients with positive anti-tTG IgA < 10× ULN had to repeat the test within 6 months. If the anti-tTG IgA value < 20 U/mL on the second test, they were excluded from the endoscopy. For patients with persistent positive anti-tTG, anti-EMA-IgA was requested.

### 2.3. Endoscopic Study

Seropositive patients were referred for endoscopy and biopsy if they accepted the procedure. Histological abnormalities associated with CD can be patchy, so at least four biopsies were taken from the distal duodenum and duodenal bulb of each patient to confirm the CD. Finally, histological interpretation of the samples was conducted by an experienced pathologist unaware of the clinical and serologic data. The results were reported according to the modified Marsh classification [21]. For diagnosis of BPCD, pathologic reports of Marsh 2 or 3 were considered diagnostic for PBCD. Marsh 2 indicates normal villi with intraepithelial lymphocytosis and crypt hyperplasia. Marsh 3 indicates intraepithelial lymphocytosis, crypt hyperplasia, and villous atrophy. Marsh 3 included three subcategories according to the severity of villous atrophy: March 3a, with mild villous atrophy, Marsh 3b, with marked villous atrophy, and March 3c, with complete villous atrophy.

When endoscopy was not accepted, we used the no-biopsy approach for the diagnosis of CD as recommended by ESPGHAN criteria for diagnosis of CD [19]. CD can be considered in symptomatic children and adolescents with anti-tTG-IgA ≥ 10× ULN and positive EMA-IgA in a second serum sample without the need for biopsy.

### 2.4. Ethical Consideration

This study was performed under the declaration of Helsinki and granted ethical approval from the Jazan Health Cluster Ethics Committee, Saudi Arabia. Participants and/or their parents gave a written informed consent on a form explaining the procedure of the study and the participant’s rights.

### 2.5. Statistical Analysis

Sociodemographic and clinical characteristics were described by descriptive analysis. Count and percentage values were calculated for categorical variables. Continuous variables that follow a normal distribution were summarized as mean ± SD, while medians represented other variables. For comparison between the seropositive and seronegative groups, the x^2^ test was used for categorical variables and the *t*-test for continuous variables. When the normality of distribution was not fulfilled, the Mann–Whitney U test was used. Statistical analyses were conducted using SPSS Version 26 (IBM, Armonk, NY, USA). The level of significance was determined at *p* < 0.05.

## 3. Results

This study included 969 participants with Type 1 diabetes, children below 14 represent 50.7% of the total sample. T1D females accounted for 59.1% of the total sample. The mean age at inclusion in this study was 14 ± 3.4 years, ranging from 2 to 18 years. The median duration of diabetes was 4 years (range 0–15 years), and the mean age at diagnosis of T1D was 9.6 ± 3.7 years (range: 1–17 years). Of the total sample, 37.2% were underweight, 44.8% had normal BMI, 11.8% were overweight, and 6.2% had obesity. Other sociodemographic and clinical features of the participants are provided in Table 1.

### 3.1. CD Seroprevalence

Anti-tTG IgA was >10× ULN in 96 patients (group 1) and <10× ULN in 68 patients (group 2). Those with Anti-tTG IgA titers < 10× ULN had to repeat the serology within 6 months. After the exclusion of 23 patients who became negative within 6 months, the final sample included 141 patients. So, the seroprevalence of CD in this study is 14.6% (141/969). Females had a higher prevalence of CD than males: 17.8% vs. 9.8%, *p* < 0.001. Serum IgA level was above the normal range in all patients. Upon review of medical records, 34 patients were known CD patients (24.1%) before inclusion in the study.

From the seropositive group, eighty-nine patients (63.1%) were diagnosed within two years of T1D diagnosis, and 29.8% were diagnosed within five years of T1D onset. The mean delay between diagnosis of T1D and CD was 3.8 ± 2.7 years. The BMI was higher in the seronegative group, 22.5 ± 4.6 kg/m^2^, compared with the seropositive, 19.1 ± 4.2 kg/m^2^ (*p* < 0.001).

Typical and atypical symptoms reported by the CD patients included gastrointestinal symptoms reported by 40.5%. The CD group had lower HbA1c (8.8 ± 2.1% vs. 9.5 ± 1.6%, *p* < 0.001) and more frequent episodes of hypoglycemia compared with T1D without CD 31.2% vs. 19.9%, *p* = 0.003. Recurrent admission due to DKA was documented in 26.3% of the CD group compared with 19.6% in the seronegative group, *p* > 0.05. About 31.9% of the seropositive group had short stature, 51.2% had vitamin D deficiency, and 41.1% had iron deficiency anemia. Other features in the CD group are presented in Table 2.

### 3.2. Histopathology Findings

**Figure 1:**
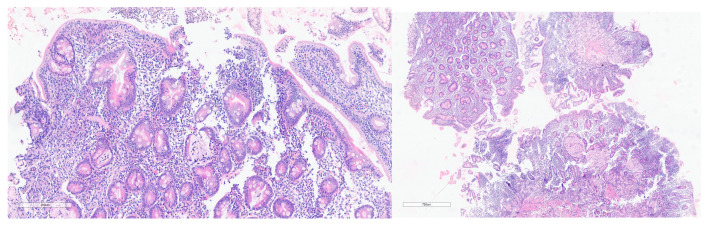
Marsh 3b with intraepithelial lymphocytosis (**left**) and marked villous atrophy (**right**).

Endoscopic examination was performed on 109 patients who accepted the procedure. Histopathological changes compatible with the diagnosis of CD were confirmed in 73 patients (Figure 1). Thus, the prevalence of biopsy-proven CD was 7.5%. Thirty-six patients had Marsh 3c, 19 patients had Marsh 3b, and 18 patients had Marsh 3a. Symptomatic patients with anti-tTG IgA above 10× ULN who refused the endoscopy were regarded as confirmed CD and started on a gluten-free diet if EMA-IgA was also positive.

## 4. Discussion

This study was conducted in the Jazan area of Saudi Arabia. Jazan is a highly populated region located in the southwestern area of Saudi Arabia. There is a growing concern about the prevalence of celiac disease in the Saudi population, especially in individuals with T1D. Understanding the relationship between these two conditions is crucial for improving the management and treatment of patients with both diseases. This study is the first study that included a large number of T1D patients from Saudi Arabia. The most important result obtained from this study is that the seroprevalence of CD was 14.6% (141/969), while the BPCD prevalence was 7.5% (73/969). CD was observed more often among females, especially those with longer diabetes duration: 17.8% vs. 9.8%, *p* < 0.001. The CD group had more frequent hypoglycemia and lower HbA1c.

Many studies using anti-tTG IgA antibody screening in different regions of Saudi Arabia have reported a high prevalence of CD in T1D patients, frequencies largely differ among reports. The reported seroprevalence of CD in T1D was 7.3%, 19.7%, and 21.2% in Jeddah, 11.5% and 24.5% in Riyadh, 10.4% in Aseer, and 7.1 in Abha [16,17,22,23,24,25,26]. A meta-analysis of eight studies in T1D Saudi patients found that 15.8% of them were seropositive for CD, while 12.8% had biopsy-proven CD [15]. This relatively high prevalence in Saudi Arabia is comparable to other studies conducted in other countries in the Arab region but much higher than what was reported in Western countries [27,28]. Recent studies from Arab countries using Anti-TTG IgA reported a CD prevalence of 9.1% in Morocco [29], 16.6% in Jordan [30], 17% in Oman [31], and only 5% in Qatar [32]. The high prevalence of diseases that have a genetic background might be related in part to the high rates of consanguinity among the Saudi population. Several studies on the Saudi population reported that consanguinity might be above 50%, and the major form is first-cousin marriages [33,34]. However, genetics and consanguinity are not the sole factors that can explain this high prevalence of T1D and CD. Different variables could be implicated, including viral infections, nutritional factors, lifestyle changes, breastfeeding practices, and exposure to different environmental pollutants and toxins [12].

One explanation for this high prevalence might be related to dietary, environmental, and genetic susceptibility shared by families in our region. The diversity of the results between different studies of Saudi Arabia could be explained by the different study designs, age of patients, sample size, genetic and geographical background, and other factors that mandate further national research from all regions of this country.

Regarding gender prevalence, our data confirm that CD is more prevalent in females with T1D (1.8:1). This is similar to other epidemiological studies over the world and in Saudi Arabia, where prevalence in females is double that in males [15,28]. Although T1D is equally prevalent among both genders in most populations, the high prevalence of CD in T1D females suggests that hidden triggers could potentiate autoimmune pathogenesis in the small intestine of the females. Some authors postulated intestinal microbiota, but the exact reasons for this CD predominance in females are still not fully understood and likely involve a combination of genetic, hormonal, and environmental factors [35].

CD can occur at any age, but individuals with younger age at diabetes diagnoses are at greater risk, especially if diabetes was diagnosed before the age of five [6,36]. However, this was not a consistent finding, as other reports found marginal differences or no differences [30,37]. In our cohort, people who have diabetes diagnosed before the age of five have a similar tendency for CD as those diagnosed with T1D above the age of five, but the longer duration of diabetes was associated with a higher prevalence of CD.

In the current study, 91.9% of CD patients were diagnosed within 5 years of T1D onset. This finding is similar to other cohorts from different studies, namely Saudi Arabia [17,22], Jordan [30], India [38], and Italy [39]. This sequential co-occurrence has traditionally been attributed to shared environmental and genetic factors, especially the HLA-DR3-DQ2 and DR4-DQ8 haplotypes [40]. Thus, we emphasize the early screening for CD in T1D within one year of diagnosis, which is recommended by the diabetes guidelines [41]. About one-third of the CD cases in this study were diagnosed after two years of diabetes onset; this also emphasizes the need for yearly screening of CD in T1D, at least for the first five years. This recommendation becomes mandatory if patients have clinical features related to CD, recurrent hypoglycemia, or uncontrolled diabetes [42].

Traditionally, patients with T1D and CD are mostly asymptomatic, while some might present with atypical features or have features related to the impact of CD on diabetes [3]. The reported prevalence of asymptomatic patients with CD and T1D ranges from 35.7% to 62.5% [8,43]. Regardless of the type of symptoms, there is often a prolonged delay between symptom onset and celiac disease diagnosis. Among the 141 patients diagnosed with CD in our study, most of them were asymptomatic or had non-specific symptoms. Gastrointestinal symptoms were reported in 40.5%, and recurrent hypoglycemia in 31.2% of the CD group. About 31.9% of CD cases have short stature, 51.2% are deficient in vitamin D, and 41.1% have iron deficiency anemia. Two surveys in Germany found that people with T1DM and CD had lower weight and height [44,45].

The American and the European guidelines for the diagnosis of CD proposed that CD could be diagnosed in symptomatic children and adolescents without the need for intestinal biopsy, provided that the level of TGA-IgA was 10-fold or more than the ULN and positive result of the EMA tests in a second blood sample [19,46]. The reliability of this no-biopsy approach for CD diagnosis was confirmed in a prospective multicenter study with a positive predictive value (PPV) of >99% [47]. In our study, the histopathological changes compatible with the diagnosis of CD were confirmed in 73 out of 109 patients who accepted the endoscopy procedure. The histopathology reports found that patients with partial or complete villous atrophy had higher levels of anti-tTG. These findings were also documented in previous studies, where the level of anti-tTG > 10× ULN had a positive correlation with the degree of villous atrophy [17,47].

In the present study, patients with CD had lower HbA1c and more frequent hypoglycemia than those with T1D alone. This impact of CD on HbA1c was more prominent in symptomatic patients who have recurrent episodes of hypoglycemia. Studies on the impact of CD on diabetes control are inconsistent [3,17,45]. Some studies reported lower HbA1c, especially in the newly diagnosed symptomatic patients with malabsorption, other studies reported higher HbA1c, especially in adults with delayed diagnosis of CD, and some studies did not find any difference in HbA1c in those with and without CD [17,48,49]. The inconsistency between results is related to different factors, including the age of patients, delay in screening for CD, presence of symptoms, the effect of gluten-free diets, and other factors that affect glycemic control [3]. Insulin requirement in the seropositive CD diabetic patients was found by some authors to be lower than that in the seronegative groups, at least before the introduction of a gluten-free diet [6,22]. However, other studies found no difference in daily insulin doses [16].

There are important limitations to consider when interpreting the findings of this study. It is important to note that future prospective national studies may provide a more comprehensive understanding of CD prevalence and associated factors in the Saudi population. This study also did not investigate the genetic background of those with or without CD, which is another limitation. Although this approach is not a recommendation for the diagnosis of CD, it will be more robust if we investigate the genetic basis of CD and T1D in this country with a high prevalence of both diseases. Additionally, future studies may consider exploring the impact of gluten-free diets on glycemic control, continuous glucose monitoring variables, and growth charts in our community.

## 5. Conclusions

This study highlighted the high prevalence of CD in Saudi patients with T1D and investigated the detrimental effect of CD on glycemic control in children and adolescents. We emphasize the importance of early screening for CD at the time of diabetes diagnosis and periodically thereafter, or if any atypical features present, especially anemia, growth delay, underweight, or frequent hypoglycemia.

## Figures and Tables

**Table 1:** Anthropometric, clinical, and biochemical characteristics.

	Seronegative (n = 828)	Seropositive (n = 141)	*p* *
Gender, males, n (%)	357 (43.1)	39 (9.8)	<0.001
Gender, females, n (%)	471 (56.9)	102 (17.8)	
Age at inclusion, years	14.9 ± 3.4	13.8 ± 3.6	<0.001
Age at T1D diagnosis, years	9.9 ± 3.7	8.1 ± 3.3	<0.001
Duration of diabetes, years	5 ± 2.9	5.7 ± 3.2	0.010
HbA1c, %	9.5 ± 1.6	8.8 ± 2.1	<0.001
BMI, kg/m^2^	22.5 ± 4.6	19.1 ± 4.2	<0.001
Anemia, n (%)	213 (25.7%)	56 (41.1)	<0.001
Short stature, n (%)	204 (24.6)	45 (31.9)	0.68
Hypoglycemia, n (%)	165 (19.9)	44 (31.2)	0.003
Recurrent DKA, n (%)	162 (19.6)	37 (26.3)	0.07
Vitamin D3 deficiency, n (%)	234 (28.3)	72 (51.1)	<0.001

* *p*-value was calculated for seropositive vs. the seronegative group.

**Table 2:** Clinical features in patients with type 1 diabetes and celiac disease.

Clinical Symptoms, n = 141	%
Abdominal pain	23.8
Diarrhea	24.1
Constipation	6.4
Anemia	41.1
Short stature	31.9
Delayed puberty	9.2
Vitamin D deficiency	51.2

## Data Availability

All the necessary information is provided within the manuscript. Any other data that support the findings of this study are available from the first author upon request.

## References

[1] El-Metwally A., Toivola P., AlAhmary K., Bahkali S., AlKhathaami A., AlSaqabi M.K., Al Ammar S.A., Jawed M., Alosaimi S.M. (2020). The Epidemiology of Celiac Disease in the General Population and High-Risk Groups in Arab Countries: A Systematic Review. BioMed Res. Int..

[2] Caio G., Volta U., Sapone A., Leffler D.A., De Giorgio R., Catassi C., Fasano A. (2019). Celiac disease: A comprehensive current review. BMC Med..

[3] Akirov A., Pinhas-Hamiel O. (2015). Co-occurrence of type 1 diabetes mellitus and celiac disease. World J. Diabetes.

[4] Ogrotis I., Koufakis T., Kotsa K. (2023). Changes in the Global Epidemiology of Type 1 Diabetes in an Evolving Landscape of Environmental Factors: Causes, Challenges, and Opportunities. Medicina.

[5] King J.A., Jeong J., Underwood F.E., Quan J., Panaccione N., Windsor J.W., Coward S., deBruyn J., Ronksley P.E., Shaheen A.A. (2020). Incidence of Celiac Disease Is Increasing Over Time: A Systematic Review and Meta-analysis. Am. J. Gastroenterol..

[6] Pham-Short A., Donaghue K.C., Ambler G., Phelan H., Twigg S., Craig M.E. (2015). Screening for Celiac Disease in Type 1 Diabetes: A Systematic Review. Pediatrics.

[7] Singh P., Arora A., Strand T.A., Leffler D.A., Catassi C., Green P.H., Kelly C.P., Ahuja V., Makharia G.K. (2018). Global Prevalence of Celiac Disease: Systematic Review and Meta-analysis. Clin. Gastroenterol. Hepatol..

[8] Camarca M.E., Mozzillo E., Nugnes R., Zito E., Falco M., Fattorusso V., Mobilia S., Buono P., Valerio G., Troncone R. (2012). Celiac disease in type 1 diabetes mellitus. Ital. J. Pediatr..

[9] Ide A., Eisenbarth G.S. (2003). Genetic susceptibility in type 1 diabetes and its associated autoimmune disorders. Rev. Endocr. Metab. Disord..

[10] Bao F., Yu L., Babu S., Wang T., Hoffenberg E.J., Rewers M., Eisenbarth G.S. (1999). One third of HLA DQ2 homozygous patients with type 1 diabetes express celiac disease-associated transglutaminase autoantibodies. J. Autoimmun..

[11] Rohrer T.R., Wolf J., Liptay S., Zimmer K.P., Fröhlich-Reiterer E., Scheuing N., Marg W., Stern M., Kapellen T.M., Hauffa B.P. (2015). Microvascular Complications in Childhood-Onset Type 1 Diabetes and Celiac Disease: A Multicenter Longitudinal Analysis of 56,514 Patients from the German-Austrian DPV Database. Diabetes Care.

[12] Robert A.A., Al-Dawish A., Mujammami M., Dawish M.A.A. (2018). Type 1 Diabetes Mellitus in Saudi Arabia: A Soaring Epidemic. Int. J. Pediatr..

[13] Al-Hussaini A., Troncone R., Khormi M., AlTuraiki M., Alkhamis W., Alrajhi M., Halal T., Fagih M., Alharbi S., Bashir M.S. (2017). Mass Screening for Celiac Disease Among School-aged Children: Toward Exploring Celiac Iceberg in Saudi Arabia. J. Pediatr. Gastroenterol. Nutr..

[14] Aljebreen A.M., Almadi M.A., Alhammad A., Al Faleh F.Z. (2013). Seroprevalence of celiac disease among healthy adolescents in Saudi Arabia. World J. Gastroenterol..

[15] Safi M.A. (2019). Celiac disease in type 1 diabetes mellitus in the Kingdom of Saudi Arabia. Characterization and meta-analysis. Saudi Med. J..

[16] Saadah O.I., Al-Agha A.E., Al Nahdi H.M., Bokhary R.Y., Bin Talib Y.Y., Al-Mughales J.A., Al Bokhari S.M. (2012). Prevalence of celiac disease in children with type 1 diabetes mellitus screened by anti-tissue transglutaminase antibody from Western Saudi Arabia. Saudi Med. J..

[17] Al-Hussaini A., Sulaiman N., Al-Zahrani M., Alenizi A., El Haj I. (2012). High prevalence of celiac disease among Saudi children with type 1 diabetes: A prospective cross-sectional study. BMC Gastroenterol..

[18] Bin-Abbas B.S., Al Qahtani M.A. (2014). Clinical guidelines for the management of type 1 diabetes in children in Saudi Arabia endorsed by the Saudi Society of Endocrinology and Metabolism, (SSEM). Int. J. Pediatr. Adolesc. Med..

[19] Husby S., Koletzko S., Korponay-Szabó I., Kurppa K., Mearin M.L., Ribes-Koninckx C., Shamir R., Troncone R., Auricchio R., Castillejo G. (2020). European Society Paediatric Gastroenterology, Hepatology and Nutrition Guidelines for Diagnosing Coeliac Disease 2020. J. Pediatr. Gastroenterol. Nutr..

[20] Girard C., De Percin A., Morin C., Talvard M., Fortenfant F., Congy-Jolivet N., Le Tallec C., Olives J.-P., Mas E. (2023). Accuracy of Serological Screening for the Diagnosis of Celiac Disease in Type 1 Diabetes Children. Medicina.

[21] Oberhuber G., Granditsch G., Vogelsang H. (1999). The histopathology of coeliac disease: Time for a standardized report scheme for pathologists. Eur. J. Gastroenterol. Hepatol..

[22] Aljulifi M.Z., Mahzari M., Alkhalifa L., Hassan E., Alshahrani A.M., Alotay A.A. (2021). The prevalence of celiac disease in Saudi patients with type 1 diabetes mellitus. Ann. Saudi Med..

[23] Al-Hakami A.M. (2016). Pattern of thyroid, celiac, and anti-cyclic citrullinated peptide autoantibodies coexistence with type 1 diabetes mellitus in patients from Southwestern Saudi Arabia. Saudi Med. J..

[24] Al-Agha A.E., Alafif M.M., Abd-Elhameed I.A. (2015). Glycemic control, complications, and associated autoimmune diseases in children and adolescents with type 1 diabetes in Jeddah, Saudi Arabia. Saudi Med. J..

[25] Alshareef M.A., Aljabri K.S., Bokhari S.A., Al Jiffri A.M., Elsaoud H.M.A., Akl A.F., Fageeh S.M., Alreffi A.N., Eltayeb A.A., Aljabri N.K. (2016). The prevalence of celiac disease in Saudi patients with type 1 diabetes mellitus: Cross sectional study. Int. J. Diabetes Metab. Disord..

[26] Alghamdi R.A., Alghamdi A.H., Fureeh A.A. (2018). Sero-prevalence of celiac disease among symptom-free type 1 diabetes mellitus in Al-Baha region, Saudi Arabia. J. Pharm. Biol. Sci..

[27] Nederstigt C., Uitbeijerse B.S., Janssen L.G.M., Corssmit E.P.M., de Koning E.J.P., Dekkers O.M. (2019). Associated auto-immune disease in type 1 diabetes patients: A systematic review and meta-analysis. Eur. J. Endocrinol..

[28] Taczanowska A., Schwandt A., Amed S., Tóth-Heyn P., Kanaka-Gantenbein C., Volsky S.K., Svensson J., Szypowska A. (2021). Celiac disease in children with type 1 diabetes varies around the world: An international, cross-sectional study of 57 375 patients from the SWEET registry. J. Diabetes.

[29] Belhiba O., Bousfiha A.A., Jennane F. (2023). Prevalence of celiac disease in Moroccan children with type 1 diabetes mellitus: A 16-year cross-sectional study. Qatar Med. J..

[30] Odeh R., Alassaf A., Gharaibeh L., Ibrahim S., Khdair Ahmad F., Ajlouni K. (2019). Prevalence of celiac disease and celiac-related antibody status in pediatric patients with type 1 diabetes in Jordan. Endocr. Connect..

[31] Al-Sinani S., Sharef S.W., Al-Yaarubi S., Al-Zakwani I., Al-Naamani K., Al-Hajri A., Al-Hasani S. (2013). Prevalence of celiac disease in omani children with type 1 diabetes mellitus: A cross sectional study. Oman Med. J..

[32] Alyafei F., Soliman A., Alkhalaf F., Sabt A., De Sanctis V., Elsayed N., Waseef R. (2018). Prevalence of β-cell antibodies and associated autoimmune diseases in children and adolescents with type 1 diabetes (T1DM) versus type 2 diabetes (T2DM) in Qatar. Acta Biomed..

[33] Warsy A.S., Al-Jaser M.H., Albdass A., Al-Daihan S., Alanazi M. (2014). Is consanguinity prevalence decreasing in Saudis?: A study in two generations. Afr. Health Sci..

[34] Khayat A.M., Alshareef B.G., Alharbi S.F., AlZahrani M.M., Alshangity B.A., Tashkandi N.F. (2024). Consanguineous Marriage and Its Association with Genetic Disorders in Saudi Arabia: A Review. Cureus.

[35] Jansson-Knodell C.L., Hujoel I.A., West C.P., Taneja V., Prokop L.J., Rubio-Tapia A., Murray J.A. (2019). Sex Difference in Celiac Disease in Undiagnosed Populations: A Systematic Review and Meta-analysis. Clin. Gastroenterol. Hepatol..

[36] Cerutti F., Bruno G., Chiarelli F., Lorini R., Meschi F., Sacchetti C. (2004). Younger age at onset and sex predict celiac disease in children and adolescents with type 1 diabetes: An Italian multicenter study. Diabetes Care.

[37] Larsson K., Carlsson A., Cederwall E., Jönsson B., Neiderud J., Jonsson B., Lernmark Å., Ivarsson S.A., Skåne Study Group (2008). Annual screening detects celiac disease in children with type 1 diabetes. Pediatr. Diabetes.

[38] Bhadada S.K., Kochhar R., Bhansali A., Dutta U., Kumar P.R., Poornachandra K.S., Vaiphei K., Nain C.K., Singh K. (2011). Prevalence and clinical profile of celiac disease in type 1 diabetes mellitus in north India. J. Gastroenterol. Hepatol..

[39] Greco D., Pisciotta M., Gambina F., Maggio F. (2013). Celiac disease in subjects with type 1 diabetes mellitus: A prevalence study in western Sicily (Italy). Endocrine.

[40] Hagopian W., Lee H.S., Liu E., Rewers M., She J.X., Ziegler A.G., Lernmark Å., Toppari J., Rich S.S., Krischer J.P. (2017). Co-occurrence of Type 1 Diabetes and Celiac Disease Autoimmunity. Pediatrics.

[41] American Diabetes Association Professional Practice Committee (2023). 14. Children and Adolescents: Standards of Care in Diabetes—2024. Diabetes Care.

[42] Fröhlich-Reiterer E., Elbarbary N.S., Simmons K., Buckingham B., Humayun K.N., Johannsen J., Holl R.W., Betz S., Mahmud F.H. (2022). ISPAD Clinical Practice Consensus Guidelines 2022: Other complications and associated conditions in children and adolescents with type 1 diabetes. Pediatr. Diabetes.

[43] DeMelo E.N., McDonald C., Saibil F., Marcon M.A., Mahmud F.H. (2015). Celiac Disease and Type 1 Diabetes in Adults: Is This a High-Risk Group for Screening?. Can. J. Diabetes.

[44] Fröhlich-Reiterer E.E., Kaspers S., Hofer S., Schober E., Kordonouri O., Pozza S.B., Holl R.W. (2011). Anthropometry, metabolic control, and follow-up in children and adolescents with type 1 diabetes mellitus and biopsy-proven celiac disease. J. Pediatr..

[45] Kaspers S., Kordonouri O., Schober E., Grabert M., Hauffa B.P., Holl R.W. (2004). Anthropometry, metabolic control, and thyroid autoimmunity in type 1 diabetes with celiac disease: A multicenter survey. J. Pediatr..

[46] Rubio-Tapia A., Hill I.D., Semrad C., Kelly C.P., Greer K.B., Limketkai B.N., Lebwohl B. (2023). American College of Gastroenterology Guidelines Update: Diagnosis and Management of Celiac Disease. Off. J. Am. Coll. Gastroenterol. ACG.

[47] Werkstetter K.J., Korponay-Szabó I.R., Popp A., Villanacci V., Salemme M., Heilig G., Lillevang S.T., Mearin M.L., Ribes-Koninckx C., Thomas A. (2017). Accuracy in Diagnosis of Celiac Disease Without Biopsies in Clinical Practice. Gastroenterology.

[48] Leeds J.S., Hopper A.D., Hadjivassiliou M., Tesfaye S., Sanders D.S. (2011). High prevalence of microvascular complications in adults with type 1 diabetes and newly diagnosed celiac disease. Diabetes Care.

[49] Hansen D., Brock-Jacobsen B., Lund E., Bjørn C., Hansen L.P., Nielsen C., Fenger C., Lillevang S.T., Husby S. (2006). Clinical benefit of a gluten-free diet in type 1 diabetic children with screening-detected celiac disease: A population-based screening study with 2 years’ follow-up. Diabetes Care.

